# Food-Drying Applications for Plant Products: A Comparative Analysis

**DOI:** 10.3390/foods12203739

**Published:** 2023-10-11

**Authors:** Nemanja Miletić, Milica Nićetin

**Affiliations:** 1 Department of Food Technology, Faculty of Agronomy, University of Kragujevac, Cara Dušana 34, 32000 Čačak, Serbia; 2 Department of Chemical Engineering, Faculty of Technology Novi Sad, University of Novi Sad, Bul. Cara Lazara 1, 21000 Novi Sad, Serbia; milican@uns.ac.rs

## 1. Introduction

Consumable plant products are seasonal and perishable items, generally only available in a fresh state for a few months each year. Therefore, the produce requires processing and/or storage at low temperatures.

Drying is one of the oldest methods of preserving plant products. Through this process, water is removed from the food matrix, resulting in a product that is microbiologically stable, with an extended shelf life and a reduced volume and mass, and thus its storage and packaging are facilitated. Drying is normally accompanied by the evaporation of water from the biological material and the transfer of moisture to the surrounding environment. The evaporation of the inner water through heat transfer is the oldest form of food drying and is still the dominant method, although it comes with certain drawbacks. However, there are many other methods for plant material drying, such as solar drying, conductive drying, convective drying, vacuum drying, freeze-drying, microwave drying, IR drying, drying in a fluidized bed, etc. [1]. Many authors have combined drying methods, accompanied by raw material pretreatment. However, every drying method causes certain undesirable physical, chemical, and biochemical transformations, such as material deformation (i.e., changes in the structure of the biological material) and degradation and reduction of bioactive substances (such as antioxidants, vitamins, and aromas) that have a positive effect on human health. On the other hand, when developing food-drying technologies, we have to consider their drying efficiency and lower their energy consumption. The drying process now needs to be accelerated, but this must not occur at the expense of the product quality [2].

Nowadays, consumers demand high-quality and additive-free products with an extended shelf life; these might be considered healthier options and even functional foods. Therefore, processors of food products of plant origin are constantly searching for drying methods, which will be either optimized traditional techniques, completely novel approaches, or synergistic combinations of several known methods.

## 2. Food Changes Caused by Drying

The type and extent of changes caused by the drying of plant material depend on the biological material itself: its composition, its physical and structural properties, etc. The changes that occur during drying are physical (e.g., changes in shape and volume, shrinking and bending, a reduction in the water content from 80–90% to about 10%, changes in sensor properties, changes in porosity, density, and pore size distribution, etc.) and chemical (e.g., reducing the water content, increasing the proportion of mineral and organic substances, reducing the contents of volatile, thermolabile and easily oxidizing aromatic substances, non-enzymatic browning, etc.) [3]. As the drying process takes longer, the degradative changes are more pronounced.

In the tissues of biological materials, water and small molecules in solution can diffuse through highly selective semi-permeable cell membranes. During the application of thermal drying processes, the damaged cell wall changes its semipermeable properties and becomes permeable to larger molecules that are dissolved in water, and the dissolved substances are concentrated in the peripheral parts of the biological material. As a consequence, degradable reactions and the hardening and shrinkage of the surface layer intensify.

Plant materials contain a number of compounds that are thermolabile, sensitive to light, and subject to oxidation. Most of these compounds offer beneficial effects for human health. The loss of these compounds is inevitable, but the extent of that loss depends primarily on the drying method and pretreatment applied. Furthermore, if high temperatures are applied in dehydration, many compounds that are not thermolabile can undergo a series of chemical reactions (including caramelization, Maillard reactions, the formation of 5-hydroxymethylfurfural, etc.) that will often lead to changes in the sensory properties of the raw material [4,5].

One of the most important quality parameters for dried foods is rehydration, which is the ability of well-dried biological material to receive the total amount of removed water and completely restore its native properties [6]. This depends on various factors, such as the type of pretreatment, the moisture content of the biological material, the method of processing, and the drying conditions. The drying method affects the density, porosity, and sorption characteristics of the product, so the choice of drying method is essential to the production of high-quality products, whether dried or rehydrated. Additionally, the drying conditions (i.e., the temperature, relative humidity, and flow rate) influence the rehydration ability. Convective drying, which has a higher drying speed at the beginning of the process, may lead to a hardening of the plant material, resulting in a lower rehydration capacity, a dense structure, and a high level of shrinkage. Microwave drying is characterized by a more porous structure, which leads to faster rehydration. The rehydration process can be improved through the use of ultrasound, which has a mechanical effect on the dried material without significantly overheating it. When vacuum drying is applied, the energy travels directly to the molecules and is then spread over the entire plant material instead of just the surface, creating a porous product [7].

Today, consumers increasingly insist on high-quality “minimally processed food”, in which the plant’s native sensorial and nutritional properties are preserved to the greatest extent due to much milder drying conditions (such as lower temperatures, a shorter processing time, the use of a vacuum, dielectric heating, or an inert atmosphere). Thus, the drying process should be strictly controlled so that, in the rehydration phase, the dried food can be used as fresh, without any significant impairment to the quality.

## 3. Pretreatment of Plant Products before Processing

In today’s world, there is a constant demand for a reduction in energy consumption in all aspects of life, primarily in industry and in food technology as its integral part. The appropriate preparation of plant products before they are dried reduces the drying time to a large extent and, consequently, reduces the energy consumption and prolongs the shelf life by decreasing the microbial load of the products. So far, various chemical preparations (immersion into a sugar or salt hypertonic solution, dipping into alkali hydroxides, exposure to ozone or CO_2_, etc.) and physical pretreatments (immersion into hot or boiling water, freezing, ultrasound treatment, microwave treatment, etc.) have been applied to plant materials [8]. Nevertheless, an increase in the processing rate, which has certainly been achieved, is not the only factor to be considered. The quality of the final product should also be increased or at least maintained. Almost all the pretreatments used in the food industry offer certain advantages but also, inevitably, some drawbacks. It is recognized that the high cost of pretreatment equipment for industrial-scale production is often a huge obstacle and means that some food pretreatments never progress beyond the laboratory stage. As for the product quality, pretreatments sometimes cause the loss of thermo-labile compounds, leading to a loss of texture, crispness, softness, and rehydration.

A compatible pretreatment–drying process, or a combination of several pretreatment–drying processes, including the modification of existing pretreatments, may offer a good solution that retains energy benefits but avoids any detrimental effects on the nutritional value of the final products.

This Special Issue aims to publish quality original research and review articles focusing on various drying methods and pretreatments for plant products (integrated and organic) in order to obtain high-quality food items via shortened low-energy drying processes.
